# Pharmacologic and Nonpharmacologic Pain Management in Patients with Traumatic Brain Injury: A Multidisciplinary Approach

**DOI:** 10.3390/jcm14248713

**Published:** 2025-12-09

**Authors:** Benjamin S. Esneault, Macie B. Maddox, Ethan M. Loewe, Miguel A. Pappolla, Tomasina Q. Parker-Actlis, Sahar Shekoohi, Alan D. Kaye

**Affiliations:** 1 School of Medicine, Louisiana State University Health Sciences Center at Shreveport, Shreveport, LA 71103, USA; 2 Department of Neurology, The University of Texas Medical Branch, Galveston, TX 77555, USA; 3 Department of Anesthesiology, Louisiana State University Health Sciences Center at Shreveport, Shreveport, LA 71103, USA

**Keywords:** traumatic brain injury, nociplastic pain, neuropathic pain, nociceptive pain, neuroinflammation, multidisciplinary management

## Abstract

Traumatic brain injury (TBI) is a major global health problem and a leading cause of long-term neurological disability. TBI produces a spectrum of persistent symptoms, including cognitive impairment, mood and behavioral disturbances, sleep disruption, fatigue, and autonomic dysregulation. Chronic pain is among the most debilitating sequelae, affecting physical, emotional, and social functioning. The etiology of post-TBI pain is multifactorial, arising from initial structural and biochemical injury to the nervous system, maladaptive neuroplastic changes, neuroinflammation, and psychological comorbidities that amplify pain perception and chronicity. This review explores the complex pathophysiology of post-TBI pain and outlines a multidisciplinary framework for management. Pain syndromes are classified according to the International Association for the Study of Pain’s mechanistic framework as nociceptive pain (resulting from tissue injury and inflammation), neuropathic pain (due to lesion or disease of the somatosensory nervous system), and nociplastic pain (arising from altered nociceptive processing without clear evidence of tissue or nerve damage). Many patients exhibit mixed pain phenotypes driven by neuroinflammation and central sensitization. Pharmacologic approaches, including anti-inflammatory agents, anticonvulsants, and antidepressants, require cautious titration due to TBI-related comorbidities. Equally essential are non-pharmacologic strategies such as physical and occupational therapy, cognitive behavioral therapy, and neuromodulation techniques, which target both biomechanical and psychosocial contributors. Emerging innovations, machine learning for prognostication, blood biomarkers for structural injury, and neuro-reparative agents, represent the next frontier in personalized management. Effective care for post-TBI pain requires an integrated strategy that combines mechanistic classification, multidisciplinary treatments, and advancing diagnostic technologies.

## 1. Introduction

Traumatic brain injury (TBI) is a leading cause of death and disability worldwide. TBI results from an external force, such as blows to the head or penetrating injuries. It is most often caused by falls or motor vehicle accidents. Rapid acceleration and deceleration of the brain without direct impact is another way TBI can occur, leading to diffuse axonal injury (DAI) [1].

In the United States, it is estimated that around 5.3 million people are living with a TBI-related disability [2]. Symptom expression varies substantially by TBI severity. Individuals with mild TBI typically experience conscious perception of pain, whereas patients with moderate to severe TBI may have impaired awareness or altered nociceptive processing in the early post-injury period. Complications of TBI include chronic pain, cognitive dysfunction, and psychological challenges [3]. Among these complications, the etiology of chronic pain is particularly complex because it concurs with common comorbidities such as post-traumatic stress disorder (PTSD), depression, anxiety, fatigue, and sleep disorders [4]. Further contributing to the complexity of TBI, neurotrauma research has several needs that remain unmet. Some of these needs include greater patient engagement, more follow-up, and greater focus on prognostication [5].

Beyond the initial mechanical injury, persistent pain in TBI often arises from maladaptive neuroplastic changes within the central nervous system that alter nociceptive processing. These maladaptive neuroplastic changes alter central nociceptive processing and form the basis for both neuropathic and nociplastic mechanisms within the International Association for the Study of Pain (IASP) framework. Neuropathic pain arises from demonstrable lesions or disease affecting the somatosensory system—such as diffuse axonal injury, thalamic dysfunction, or disruption of descending inhibitory circuits—resulting in burning, shooting pain and sensory abnormalities. In contrast, nociplastic pain develops in the absence of clear structural damage, emerging instead from altered nociceptive signaling characterized by central sensitization, impaired inhibitory control, and heightened neural responsivity to non-noxious stimuli. Together, these neuropathic and nociplastic processes reflect the continuum of central sensitization following TBI and contribute to the mixed and often overlapping pain phenotypes observed in many patients [3,6]. To mitigate these changes, a primary focus of acute TBI care must be the prompt and adequate treatment of pain to prevent central sensitization from becoming entrenched, thus avoiding the development of long-term chronic pain syndromes.

The imbalance between excitatory and inhibitory modulation, characterized by impaired descending pain inhibition and hyperexcitability of central nociceptive circuits, leads to hypersensitivity and overlapping pain phenotypes [6]. Consequently, pain after TBI is multifactorial, encompassing nociceptive mechanisms from peripheral injury, neuropathic pain due to tract or thalamic damage, and nociplastic mechanisms associated with cortical dysregulation and neuroinflammation [7].

Because of this mechanistic diversity, effective treatment of chronic pain following TBI requires an integrated, multidisciplinary approach that combines pharmacologic and non-pharmacologic interventions. Post-traumatic headache (PTH), the most common pain sequela of TBI, illustrates this complexity: while simple analgesics such as NSAIDs or paracetamol may suffice in mild cases, persistent pain often requires multimodal management involving rehabilitation, behavioral therapy, and psychological support [8]. Such an approach, uniting neurologic, psychological, and rehabilitative perspectives, extends beyond headache to address the broad spectrum of pain and disability following TBI.

The current review, therefore, explores the mechanisms behind post-TBI pain, including nociceptive, neuropathic, and nociplastic factors, and highlights the multidisciplinary strategies necessary for its treatment. This narrative review was conducted through a comprehensive literature search using PubMed. Searches were performed using combinations of the following terms: traumatic brain injury, chronic pain, nociceptive pain, neuropathic pain, nociplastic pain, post-traumatic headache, central sensitization, rehabilitation, pharmacologic management, non-pharmacologic management, cognitive behavioral therapy, transcranial magnetic stimulation, interdisciplinary pain rehabilitation, CGRP, artificial intelligence, biomarkers, and machine learning. Additional articles were identified through reference lists of key publications. Priority was given to peer-reviewed studies, clinical guidelines, meta-analyses, and major review articles.

## 2. Pathophysiology of Pain in Traumatic Brain Injury

Pain following TBI can be understood within the framework of the IASP pain classification, which includes nociceptive, neuropathic, and nociplastic pain mechanisms. Most patients experience mixed pain types that reflect overlapping peripheral and central processes.

Nociceptive (musculoskeletal) pain arises from tissue or musculoskeletal injury, spasticity, or abnormal posture. It usually presents as aching, throbbing, or soreness immediately after trauma. A common symptom is neck or cervical pain, reported in about two-thirds of patients within 72 h of injury and persisting in nearly half after 45 days [9]. Noxious stimulation of peripheral tissues activates nociceptive afferents through ligand- and voltage-gated ion channels [2,10]. These nociceptors also interact with immune mediators, emphasizing the close connection between inflammation and pain. Spasticity, which is sustained involuntary muscle contraction, further contributes to nociceptive pain and affects 30–50% of individuals with TBI [11]. Studies have demonstrated that, even in patients with severely impaired consciousness, noxious stimulation elicits measurable behavioral, autonomic, and cortical responses in the acute and early post-injury phases. This highlights that nociception may be preserved even in the absence of overt conscious pain perception, meaning that although a patient cannot consciously feel pain, the nervous system is still reacting to noxious (painful) stimuli. [12].

Neuropathic pain results from direct injury to central somatosensory pathways and usually appears as burning, shooting pain, paresthesia, allodynia, or hyperalgesia. The spinothalamic tract (STT), a primary ascending pathway that transmits pain and temperature, is often affected, and its damage is linked to central pain in TBI [13]. The thalamus is also susceptible to hypoperfusion and structural injury, which leads to impaired ascending transmission and a loss of descending inhibitory control [14]. Further damage to the periaqueductal gray (PAG), a midbrain center vital for descending modulation, worsens this imbalance [15]. Disruptions in cortical regions like the prefrontal and insular cortices, as well as limbic structures such as the amygdala and nucleus accumbens, impair the emotional and cognitive regulation of pain [16,17]. These lesions cause central neuropathic pain, characterized by persistent hypersensitivity and abnormal pain perception related to identifiable injury within the central nervous system.

In addition to structural lesions, nociplastic pain mechanisms frequently emerge after TBI [18]. Nociplastic pain results from altered nociceptive processing without clear evidence of ongoing tissue injury or nerve lesion. Nociplastic pain also reflects dysfunction within cortical pain-modulating regions, including the dorsolateral prefrontal cortex and anterior insula, which contributes to altered salience, impaired descending inhibition, and amplification of pain perception even in the absence of structural injury. It is closely linked to central sensitization, a state of hyperexcitability of second-order neurons within the dorsal horn and higher centers that amplifies pain signaling [16]. Central sensitization bridges the neuropathic and nociplastic domains and helps explain the persistence and generalization of pain in many TBI survivors.

Post-traumatic headache (PTH) is the most common symptom of post-TBI pain and can feature both nociceptive and neuropathic (or nociplastic) mechanisms. In this review, PTH refers to the clinical headache syndrome that emerges within 7 days of injury or regaining consciousness, consistent with ICHD-3 criteria, and includes both the acute (<3 months) and chronic (>3 months) forms. Its pathophysiology includes dysfunction of the trigeminovascular system, cortical spreading depolarization, ionic fluxes, axonal injury, and altered brain metabolism [19]. Disruption of the blood–brain barrier (BBB) and subsequent neuroinflammation further enhance nociceptive and neuropathic signaling by increasing permeability and releasing cytokines [20]. BBB disruption facilitates the release of pro-inflammatory cytokines and neuropeptides, most notably calcitonin gene-related peptide (CGRP), into perivascular and trigeminal pathways. This surge in inflammatory mediators amplifies trigeminovascular activation and nociceptor sensitization, a key mechanism driving the development and chronification of PTH. These processes sensitize trigeminal afferents and central pain pathways, which contributes to headache chronicity [21]. Clinically, PTH may resemble primary headache disorders like migraine or tension-type headache, or present as cervicogenic headache when involving pericranial and cervical structures. Evidence from interventional studies, including occipital and trigeminal nerve blocks, indicates that modulating these peripheral inputs can break the cycle of central sensitization and significantly relieve symptoms in refractory cases [22].

**Figure 1:**
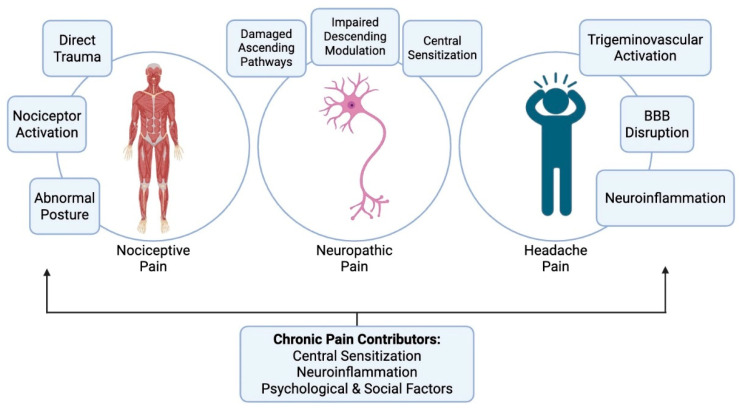
The major types of pain in TBI, their primary mechanisms, and the factors that cause these pains to persist chronically.

Thus, post-TBI pain involves a combination of nociceptive, neuropathic, and nociplastic mechanisms. Neuroinflammation and central sensitization serve as common processes that drive the transition from acute injury-related pain to chronic and often disabling pain. A summary of the major types of pain in TBI is illustrated in Figure 1.

## 3. Pharmacologic Management

Pharmacologic management of pain following TBI must address the complex and overlapping mechanisms underlying nociceptive, neuropathic, nociplastic, and headache pain. The treatment approach is often multimodal, integrating several classes of medications that act on distinct pathways to provide symptom relief while minimizing side effects.

Nonsteroidal anti-inflammatory drugs (NSAIDs) and acetaminophen are commonly used first-line agents for musculoskeletal and headache pain due to their efficacy in reducing inflammation and nociceptive transmission [23]. NSAIDs inhibit cyclooxygenase (COX) enzymes, reducing prostaglandin synthesis and peripheral sensitization, while acetaminophen acts centrally to inhibit prostaglandin production and modulate serotonergic descending pathways [24]. However, chronic NSAID use carries gastrointestinal, cardiovascular, and renal risks, particularly in older or medically complex TBI patients [23]. It can also exacerbate headaches through rebound mechanisms.

Tricyclic antidepressants (TCAs) such as amitriptyline and nortriptyline are frequently used for neuropathic and post-traumatic headache (PTH) pain [25]. Their analgesic effects derive from inhibition of serotonin and norepinephrine reuptake in descending pain pathways, enhancing endogenous pain inhibition [25]. TCAs may also benefit comorbid depression, anxiety, and sleep disturbances, which are common after TBI [25]. However, their anticholinergic effects can worsen cognition, memory, and alertness, problems frequently encountered in TBI patients. Therefore, doses should be kept low and titrated cautiously, balancing analgesic benefit with cognitive safety. TCAs should also be used carefully in patients with cardiac conduction abnormalities or orthostatic hypotension [26].

Serotonin-norepinephrine reuptake inhibitors (SNRIs) such as duloxetine and venlafaxine are effective for neuropathic pain syndromes. A meta-analysis recommends duloxetine, venlafaxine, and extended-release gabapentin as first-line agents for neuropathic pain based on GRADE criteria [27]. SNRIs are often preferred to TCAs for TBI patients due to a better cognitive and cardiovascular safety profile [27].

Norepinephrine-specific reuptake inhibitors (NRIs) such as reboxetine or atomoxetine may also be useful for pain modulation through enhanced activation of descending noradrenergic inhibitory pathways [28]. Although less studied in TBI populations, NRIs can be considered when serotonergic drugs are poorly tolerated or contraindicated, with attention to potential increases in blood pressure and heart rate [29].

Anticonvulsants such as gabapentin and pregabalin are cornerstone agents for neuropathic pain after TBI [30]. Both act by binding the α2δ subunit of voltage-gated calcium channels, reducing excitatory neurotransmitter release and neuronal hyperexcitability. Pregabalin offers more predictable pharmacokinetics and may yield greater pain reduction and improved sleep quality compared to gabapentin, with comparable tolerability [31]. In TBI, these agents may also stabilize mood and improve sleep while reducing neuropathic pain intensity [30].

Muscle relaxants, including baclofen and tizanidine, are used primarily for spasticity-related nociceptive pain [11]. Baclofen acts as a GABA-B receptor agonist to inhibit spinal excitatory neurotransmission, whereas tizanidine functions as an α2-adrenergic agonist to reduce muscle tone [32]. Both can alleviate painful spasms and improve range of motion, though sedation, hypotension, and dizziness can limit their use [32].

Beta-blockers, particularly propranolol, are sometimes used in post-traumatic headache management because they blunt sympathetic nervous system activity by reducing circulating catecholamines [33]. Propranolol should be avoided in patients with asthma, bradycardia, or hypotension [34]. Beyond headache control, beta-blockers may also confer cardiovascular benefits and improved in-hospital outcomes in TBI [33].

Acute headache treatments include triptans, serotonin 5-HT1B/1D receptor agonists that cause vasoconstriction of cranial vessels and suppress release of CGRP and other pro-inflammatory neuropeptides [35]. Triptans are most effective for migraine-like PTH but are reserved for acute rather than preventive use [36]. Failure to treat acute episodes adequately may lower the migraine threshold, leading to attack recurrence and chronicity [36].

Prophylactic headache treatments are key to reducing PTH frequency and severity. Medications like topiramate, valproic acid, and other antiepileptics may be used for migraine-like PTH, but contraindications should be considered [37,38]. For instance, topiramate should be avoided in patients with nephrolithiasis, metabolic acidosis, or cognitive slowing. CGRP monoclonal antibodies (e.g., erenumab, fremanezumab, galcanezumab, eptinezumab) mark an important advancement for chronic migraine-like headaches and might be options in refractory PTH cases, although data specific to TBI populations are still emerging [39]. These antibodies block CGRP-mediated neurogenic inflammation and trigeminovascular activation, which are involved in PTH pathophysiology.

Botulinum toxin type A has demonstrated efficacy for chronic headache and focal spasticity after TBI [40]. It inhibits acetylcholine release at the presynaptic cleft, reducing neuromuscular transmission and pain from muscle hyperactivity [32]. Before initiating treatment, trigger factors and contractures should be addressed, and physiotherapy, splinting, or casting may enhance benefit [32].

Interventional pain procedures may be considered when conservative therapy fails [22]. Cervical facet joint interventions, including diagnostic medial branch blocks and radiofrequency ablation, can provide relief in cervicogenic headaches linked to whiplash-associated or post-traumatic neck injury. Peripheral nerve blocks, such as occipital, supraorbital, or supratrochlear blocks, are effective abortive or adjunctive options for post-traumatic migraine and cervicogenic headache, particularly when combined with pharmacologic prophylaxis or neuromodulatory rehabilitation [22].

Other agents, including corticosteroids and opioids, have limited and situation-specific use. Corticosteroids may temporarily suppress inflammation but are not suitable for long-term management because of systemic side effects [41]. Opioids such as morphine can alleviate severe pain and help modulate dysautonomia in select patients but carry high risks of dependency, sedation, and respiratory depression [42,43].

In conclusion, pharmacologic management after TBI requires careful titration, frequent reassessment, and integration with non-pharmacologic and interventional modalities to address the multifactorial and mechanistically diverse nature of post-injury pain. The mechanisms of action and main drawbacks of each class are summarized in Table 1.

## 4. Non-Pharmacologic Management

The multidisciplinary approach to pain management in TBI is a combination of both pharmacologic and non-pharmacologic management. Effective long-term management of post-TBI pain necessitates a paradigm shift from simple analgesic prescriptions toward an individualized, patient-centered approach that targets the mechanisms of chronic pain using a multitude of strategies. Non-pharmacologic strategies play a key role in addressing the maladaptive neuroplastic and nociplastic mechanisms underlying chronic pain after TBI, promoting cortical reorganization and functional recovery.

Non-pharmacologic management strategies of TBI include physical therapy, occupational therapy, cognitive-behavioral therapy (CBT), and transcranial magnetic stimulation (TMS). After moderate to severe TBI, patients are typically admitted to the ICU and later treated with extensive rehabilitation due to risk of neuromuscular complications [48]. This rehabilitation is provided by a team of rehabilitation specialists including physiatrists, neurologists, and therapists [48]. Physical therapy is considered a multimodal pain treatment because it consists of multiple mechanisms of action and is therefore effective in treating all types of pain in TBI [49]. Physical therapists (PTs) treat musculoskeletal pain using manual therapy that relieves pain, increases joint range of motion, and improves function [49]. For neuropathic pain, exercise therapy has proven to be a disease-modifying treatment by promoting healing of injured tissues [49]. Additionally, mobilization, a type of manual therapy, has been shown potential to be an effective remedy for neuropathic pain in animal and cadaver studies, but further research is needed [49]. One role of physical therapists is to evaluate movement dysfunction [49].

Movement dysfunction often presents because of pain and can appear as either motor weakness or increased muscle tension. The approach to treatment depends on whether the dysfunction is a direct result of pain or an adaptation to pain [49]. Occupational therapists (OTs) are another essential part of the rehabilitation team in TBI. OT interventions to treat chronic pain include training components such as graded activity, pacing, energy conservation strategies, environmental modifications and ergonomics related to activities at home as well as at work [50]. PTs and OTs are essential members of interdisciplinary pain rehabilitation (IPR) programs. IPR combines CBT-based pain coping skills training, graded exercise therapy, occupational and physical rehabilitation, and medication optimization within a coordinated biopsychosocial framework. While the primary aim of IPR focuses on improving functioning and well-being in spite of pain, patients who receive IPR often show improvement with both their disabilities and pain. This improvement can be attributed to the idea that IPR plays a role in desensitizing the central nervous system [51]. Optimal outcomes are achieved when physical, occupational, and psychological therapies are coordinated under a multidisciplinary rehabilitation plan, ensuring that behavioral conditioning complements physical re-training and cognitive recovery.

Another component of non-pharmacologic management of TBI pain is CBT. Importantly, CBT for chronic pain is not simply a treatment for anxiety or mood symptoms; it actively modulates pain perception. A resting-state connectivity study showed that CBT changes intrinsic connectivity between prefrontal cortex, amygdala, and PAG in chronic pain [52]. Studies have shown the use of CBT and other modalities such as relaxation techniques, biofeedback, and psychoeducation for the treatment of PTH pain [53]. A recent article showed that CBTs and multimodal and/or interdisciplinary programs have demonstrated impressive outcomes including headache-related disability, pain intensity and quality of life [54]. Research findings suggest that using approaches that include pharmacologic and non-pharmacologic interventions and manage both the physiological and psychological dimensions of persistent PTH is an optimal route for PTH treatment [54]. Recent studies also support the integration of neurofeedback and virtual-reality–assisted rehabilitation, which use real-time brain or motion feedback to enhance neuroplasticity and reduce pain perception. Graded motor imagery and mirror-based training have shown early promise for cortical reorganization in patients with neuropathic and nociplastic pain.

TMS is a noninvasive therapy originally used for major depressive disorder, and studies have investigated its use for chronic pain in TBI [55]. Repetitive TMS therapy has been shown to improve headache symptoms and overall pain in patients with TBI; however, the evidence is inconsistent and sample sizes have generally been small [55]. Repetitive TMS targeting the dorsolateral prefrontal cortex and motor cortex has shown modest efficacy for pain modulation and mood improvement, while transcranial direct current stimulation (tDCS) is under investigation for augmenting cortical connectivity and symptom control. TMS is widely considered to be safe, so it is a viable treatment option for chronic pain in TBI patients who have exhausted other options [55].

Other non-pharmacologic modalities include biofeedback, acupuncture, and relaxation therapies. Biofeedback is a form of operant conditioning designed to increase awareness of the body by modulating a person’s physiology to improve control over emotions and thoughts [56]. Studies on biofeedback have shown a decrease in TBI pain, particularly with headache [56]. Acupuncture uses needles or other nonpenetrating methods to stimulate specific energetic points on the body and could potentially help alleviate pain via reduced stress and anxiety [56]. Acupuncture should be considered for management of posttraumatic headache, though future investigations are needed to evaluate efficacy [53]. Together, these non-pharmacologic modalities complement pharmacologic and interventional treatments by addressing the affective, cognitive, and nociplastic components of pain identified in the IASP mechanistic model.

## 5. Emerging Trends in TBI Pain Management

The medical field of pain management is a rapidly evolving specialty that involves diagnosing, treating, and relieving both acute and chronic pain through a multidisciplinary approach [57]. Ongoing research and discussions in this area are essential for enhancing patient care and outcomes. New and improved healthcare methods are constantly emerging, especially related to treating pain in patients with TBI. This includes both pharmacologic and nonpharmacologic strategies. Emerging approaches aim not only to address symptoms but also to directly target the underlying pathophysiologic processes, neuroinflammation, maladaptive plasticity, and central sensitization that sustain chronic pain after TBI.

Recent advances in medically relevant artificial intelligence could open new possibilities for pain monitoring and modulation. One way AI is being used to enhance patient care is through machine learning [58]. The relationship between machine learning and TBI management is a rapidly developing field. The advantage of machine learning here is the ability to process numerous variables and perform statistical analysis that is believed to surpass traditional human-led analysis in understanding complex issues like brain injury [58]. One study using machine learning aimed to predict suicidal ideation, a potentially fatal outcome following TBI [59]. This study employed a 10-fold cross-validation gradient boosting machine analysis that showed excellent classification performance with an accuracy of 0.78 (95% confidence interval 0.77–0.79) [59]. Such a precise predictive model can aid physicians in planning and providing preventative care for their patients. Another application of AI-powered machine learning involves predictive models for post-operative outcomes. In one study, these models were found to deliver rapid and accurate predictions to guide surgical and medical decision-making [60]. Quick, reliable, and precise tools are essential for diagnosis and treatment after traumatic injuries. These predictive models can also reassure physicians about their treatment choices. Additionally, AI-driven pattern recognition may help classify patients by pain type, nociceptive, neuropathic, or nociplastic, facilitating more specific therapeutic approaches.

One of the first decisions a physician makes when a patient shows signs of a potential brain injury from head trauma is whether to order a computed tomography (CT) scan. This critical decision process is often inefficient, as 90–95% of scanned patients show no abnormalities and were only exposed to radiation [61]. Detecting structural damage earlier could help clinicians customize analgesic and neuroprotective treatments based on the underlying injury rather than relying solely on symptoms. With new criteria being adopted, such as blood biomarkers, physicians can enhance prognostic decision-making for patients with normal CT scans. Elevated levels of certain blood biomarkers in patients with head injuries and normal CT results suggest the presence of structural brain damage [62]. Most of these blood-based protein biomarker tests are currently used only in research. The clinical importance of these tests underscores the need for further research to bring them into routine hospital use. Beyond diagnosis, biomarkers like GFAP, UCH-L1, NfL, and Tau might also offer insights into neuroinflammation and long-term pain susceptibility. One new criterion that has emerged that includes biomarkers and other essential components of TBI is the CBI-M framework. This proposed new framework was created for the characterization of acute TBI and incorporates four pillars: a clinical pillar (full GCS and pupillary reactivity); a biomarker pillar (blood-based measures); an imaging pillar (pathoanatomical measures); and a modifier pillar (features influencing clinical presentation and outcome; CBI-M). Although this framework requires refinement and validation before implementation into clinical practice, it takes a step in the right direction for improving the characterization of TBI [63].

Another recent area of interest is peripheral nerve repair. Peripheral nerve injury often results from mechanical trauma and can lead to a range of issues for the patient, including neuropathic pain and motor and sensory deficits. Autologous nerve implantation has traditionally been the preferred method of repair but comes with patient-specific limitations [64]. Recent advancements using peripheral blood mononuclear cells (PBMCs) have shown promise in promoting nerve regeneration and neuro-reparative properties [64]. These cells create an enriching environment conducive to repair. Further research into PBMCs is necessary to incorporate them into routine practice. Vitamin B12 has long been recognized as vital for nerve health and function.

It is essential for myelin and neurotransmitter synthesis and provides protection against oxidative stress [65]. One study aimed to determine whether vitamin B12 could improve outcomes in TBI patients. The findings suggested that vitamin B12 supports neurological recovery by downregulating endoplasmic reticulum stress-related apoptotic pathways [66]. Additionally, vitamin B12 enhances microtubule stabilization and remyelination of peripheral nerves, which may prove beneficial as a neuroprotective agent after TBI [66].

New frontiers in neuromodulation, including vagus nerve stimulation and noninvasive cortical stimulation, aim to modulate central pain circuits and autonomic imbalance after TBI. Parallel advances in regenerative medicine, such as bioengineered nerve scaffolds and stem-cell-derived exosomes, offer potential for restoring axonal integrity and reducing chronic pain. Ultimately, the integration of AI-guided diagnostics, biomarker-based monitoring, and regenerative or neuromodulatory therapies signifies a shift toward precision pain medicine in TBI, combining biological repair with functional rehabilitation.

## 6. Discussion

TBI is a common public health issue that occurs worldwide with long-term effects on patients and families. This narrative review examines the core concepts of TBI, pharmacologic management, non-pharmacologic strategies, and recent advances in pain management related to this complex injury. This paper discusses how TBI is a unique condition that requires a multidisciplinary approach for adequate care.

Pain following TBI often involves multiple overlapping mechanisms. According to the International Association for the Study of Pain (IASP) classification, post-TBI pain can be categorized into nociceptive, neuropathic, and nociplastic types, although most patients display mixed phenotypes [4]. Nociceptive pain stems from peripheral tissue or musculoskeletal injury, neuropathic pain results from lesions in central somatosensory pathways, and nociplastic pain arises from altered nociceptive processing and central sensitization. The interplay of these mechanisms, especially the role of central sensitization in maintaining neuropathic pain and increasing nociceptive and headache-related pain, forms the basis for a multimodal, mechanism-based management approach [16,22].

Pharmacologic management must account for this mechanistic diversity while balancing efficacy and tolerability in a neurologically vulnerable population. NSAIDs remain first-line agents for nociceptive and inflammatory pain, but their gastrointestinal, cardiovascular, and renal side effects limit long-term use [23]. Acetaminophen continues to be useful in mild to moderate cases but requires caution in patients with hepatic dysfunction [24]. Tricyclic antidepressants, particularly amitriptyline and nortriptyline, are effective for neuropathic and headache-related pain, though doses must be kept low to avoid anticholinergic cognitive effects, which can exacerbate the cognitive deficits already present after TBI [25]. The introduction of serotonin-norepinephrine reuptake inhibitors (SNRIs), such as duloxetine and venlafaxine, and norepinephrine reuptake inhibitors (NRIs) like reboxetine or atomoxetine, provides effective alternatives with a more favorable safety profile [27].

Anticonvulsants such as gabapentin and pregabalin are now cornerstones in the treatment of neuropathic and nociplastic pain, improving pain intensity and sleep regulation while stabilizing mood [30,31]. Beta-blockers (especially propranolol) remain a mainstay for post-traumatic headaches related to their modulation of sympathetic overactivity [33,34].

A newer generation of beta-blockers has emerged with improved pharmacodynamic properties and fewer systemic side effects. Third-generation beta-blockers, such as nebivolol, combine β1-selective antagonism with nitric oxide–mediated vasodilation (which may improve cerebral hypoperfusion in TBI), providing effective migraine and headache prevention while reducing adverse respiratory and metabolic effects. Unlike non-selective first-generation agents, nebivolol does not worsen bronchospasm, making it safer for patients with asthma or chronic obstructive pulmonary disease, and it avoids increasing insulin resistance or dyslipidemia, limitations of earlier beta-blockers [34].

For refractory headache prevention, topiramate, valproic acid, and other antiepileptics offer prophylactic benefit, with attention to contraindications such as nephrolithiasis and cognitive slowing. The development of calcitonin gene-related peptide (CGRP) monoclonal antibodies, erenumab, fremanezumab, galcanezumab, and eptinezumab, represents a major therapeutic advancement for migraine-like post-traumatic headache. Triptans remain reserved for acute management, whereas botulinum toxin type A is valuable in chronic headache and spasticity management [35,36]. For cervicogenic or refractory migraine-type headaches, cervical facet interventions and peripheral nerve blocks such as occipital or supraorbital blocks provide effective adjunctive relief when pharmacologic therapies are insufficient.

An important clinical consideration in managing post-traumatic headache with medication is the risk of rebound or medication-overuse headache. Frequent use of acute analgesics, especially combination analgesics, opioids, triptans, or NSAIDs, can paradoxically increase headache frequency and severity. To minimize this risk, acute pain medications should typically be limited to no more than two days per week, while preventive strategies and non-pharmacologic interventions are optimized to ensure long-term control.

Another current gap in management lies in the underutilization of non-pharmacologic strategies. Patients often rely heavily on medication, overlooking interventions such as physical and occupational therapy, cognitive behavioral therapy (CBT), transcranial magnetic stimulation (TMS), biofeedback, acupuncture, and relaxation therapy, all of which target neuroplastic and psychosocial aspects of chronic pain [49,50,53,54,55,56]. These therapies are most effective when integrated under a multidisciplinary framework that combines neurorehabilitation, behavioral modification, and patient education to address both biological and emotional components of chronic pain. A crucial element of TBI pain management is recognizing that early, aggressive treatment of acute pain is fundamentally a preventative strategy. By disrupting neuroinflammation and central sensitization in the acute phase, the management team can proactively reduce the likelihood of the patient developing a disabling chronic pain condition. Among the members of the care team who play an essential role in this timely management are nurses. While physicians and physical therapists are key, nurses are on the frontline of care, providing continuous, real-time pain assessment and administering time-sensitive interventions. Their expertise in patient education is crucial. Furthermore, the multidisciplinary approach to pain in TBI would be incomplete without neuropsychologists, who play an essential role in addressing the cognitive, emotional, and behavioral components of pain. By treating co-morbidities like depression, anxiety, and post-traumatic stress, and by providing strategies such as CBT, they facilitate better pain coping mechanisms and increase the efficacy of combined treatment. Finally, the importance of strong support systems for TBI patients is critical to acknowledge. Effective treatment plans must be simplified and reinforced by family members and caregivers, who should be educated on medication safety, pain identification, and non-pharmacological techniques. Shifting the focus from a purely patient-centric model to a patient-and-family-centric approach is highly relevant, ensuring treatment is not only prescribed but also successfully implemented and maintained outside the clinic. This shared responsibility improves adherence, prevents medication misuse, and ultimately leads to better long-term functional recovery.

The emergence of artificial intelligence (AI) and machine learning is setting the stage for a new era in TBI management. AI-based systems have demonstrated the ability to identify correlations among symptom patterns, neurophysiological data, and treatment outcomes, thereby facilitating mechanism-based care tailored to individual pain phenotypes [58]. Blood biomarkers such as GFAP, NF-L, Tau, and UCH-L1 add diagnostic precision by identifying axonal, astrocytic, and neuronal injury [62,67]. The integration of these biomarkers with predictive AI models may soon allow clinicians to move beyond symptom-based management toward precision pain medicine that aligns treatments with mechanistic subtypes of TBI-related pain.

Continued research into neuroregenerative and neuromodulatory strategies also holds promise. Peripheral blood mononuclear cells (PBMCs) and vitamin B12 supplementation have demonstrated neuro-reparative effects in early studies [64,65,66]. Similarly, innovations in vagus nerve stimulation, transcranial direct current stimulation (tDCS), and bioengineered nerve scaffolds aim to restore cortical balance and reduce chronic central sensitization. These therapies, combined with AI-guided analytics and biomarker monitoring, will likely define the next frontier of multimodal TBI pain care.

## 7. Conclusions

This narrative review emphasizes the need for a multidisciplinary and mechanistically informed approach to TBI pain management. Recognizing the complex interplay between nociceptive, neuropathic, and nociplastic mechanisms, clinicians must integrate pharmacologic, interventional, and rehabilitative strategies to achieve optimal outcomes. Pharmacologic treatments, ranging from anticonvulsants, antidepressants, and beta-blockers to emerging agents such as CGRP monoclonal antibodies, should be used judiciously, accounting for both efficacy and cognitive safety. Non-pharmacologic modalities, including rehabilitation therapies, CBT, and neuromodulation, complement these treatments by targeting neuroplastic recovery and psychological resilience.

Future directions in post-TBI pain management will increasingly rely on the convergence of biomarker-based injury characterization and AI-assisted pain phenotyping. Blood biomarkers such as GFAP, UCH-L1, NfL, and Tau provide objective insight into neuronal injury, while machine-learning models can integrate these data with clinical features to classify patients by mechanistic pain subtype. Together, these tools offer the strongest opportunity to move beyond symptomatic treatment and toward an etiological, mechanism-based framework capable of addressing the specific nociceptive, neuropathic, and nociplastic processes driving chronic post-TBI pain. As the field progresses, collaboration between neuroscientists, clinicians, and rehabilitation specialists will be essential to translating these innovations into effective, evidence-based protocols. Through this integrative and evolving framework, clinicians can better address the multifaceted nature of post-TBI pain and advance toward truly personalized recovery pathways.

## Figures and Tables

**Table 1:** Drug classes, mechanisms of action, and drawbacks of pharmacologic interventions used to treat pain and headache following traumatic brain injury (TBI).

Drug/Class	Drug Class	Pain Treated	Mechanism of Action	Drawbacks/Precautions
Morphine	Opioid	Nociceptive	Modulates central nervous system pathways; may reduce sympathetic hyperactivity and exert anti-inflammatory effects through opioid receptor activation [42,43]	Respiratory depression, dependence, sedation, nausea, constipation, hypotension, histamine release, and abuse potential [42]
NSAIDs	Anti-inflammatory	Nociceptive/Headache	Inhibit COX-1 and COX-2 enzymes, reducing prostaglandin synthesis and peripheral sensitization [23]	Gastric mucosal injury, renal impairment, cardiovascular risk, hepatotoxicity, bleeding tendency [44]
Acetaminophen	Analgesic	Nociceptive/Headache	Inhibits central prostaglandin synthesis and modulates serotonergic descending pathways [24]	Hepatotoxicity at high doses, nephrotoxicity, cardiovascular risk [24]
Corticosteroids	Corticosteroid	Nonspecific	Suppress pro-inflammatory cytokine signaling and immune activation [41]	Hyperglycemia, osteoporosis, infection risk, hypertension, mood changes, gastrointestinal bleeding [41,45]
Gabapentin	Antiepileptic	Neuropathic	Binds to α2δ subunit of voltage-gated calcium channels; decreases excitatory neurotransmitter release [30]	Sedation, dizziness, peripheral edema, confusion [30]
Pregabalin	Antiepileptic	Neuropathic	Similar to gabapentin; reduces calcium influx and neurotransmitter release, improving neuropathic pain and sleep [31]	Somnolence, dizziness, weight gain, blurred vision, dependence risk [31]
TCAs (amitriptyline, nortriptyline)	Antidepressant	Neuropathic/Headache	Inhibit presynaptic reuptake of norepinephrine and serotonin, enhancing descending pain inhibition [25,46]	Anticholinergic effects (dry mouth, constipation, urinary retention, cognitive impairment), sedation, cardiac conduction abnormalities; doses must be minimized in TBI to avoid cognitive decline [26]
SNRIs (duloxetine, venlafaxine)	Antidepressant	Neuropathic	Block reuptake of serotonin and norepinephrine, augmenting descending inhibitory control [27,46]	Hypertension, nausea, insomnia, dry mouth, sexual dysfunction [27]
NRIs (reboxetine, atomoxetine)	Antidepressant	Neuropathic	Selectively inhibit norepinephrine reuptake to enhance descending noradrenergic pain modulation [28]	Increased heart rate, hypertension, insomnia, anxiety; limited TBI data [29]
SSRIs	Antidepressant	Nonspecific	Enhance serotonergic transmission; may help mood and comorbid anxiety [40,46]	Emotional blunting, weight gain, sexual dysfunction [47]
Baclofen	GABA agonist	Nociceptive	GABA-B receptor agonist; inhibits spinal excitatory neurotransmission, reducing spasticity [32]	Drowsiness, weakness, dizziness, urinary incontinence, sexual dysfunction [32]
Tizanidine	α2-adrenergic agonist	Nociceptive	Inhibits presynaptic motor neuron excitation and increases central noradrenergic tone [32]	Sedation, dry mouth, hypotension, hepatic enzyme elevation [32]
Beta-blockers (propranolol)	β-adrenergic receptor blocker	Headache	Decrease sympathetic tone and catecholamine release; preventive therapy for post-traumatic headache [33]	Bradycardia, hypotension, bronchospasm, depression, vivid dreams [34]
Topiramate	Antiepileptic/Migraine prophylactic	Headache	Enhances GABA activity, blocks voltage-gated sodium channels, inhibits glutamate receptors and carbonic anhydrase [37]	Cognitive slowing, paresthesia, kidney stones, metabolic acidosis, weight loss; avoid in nephrolithiasis or cognitive dysfunction [37]
Valproic acid/Divalproex	Antiepileptic/Headache prophylactic	Headache	Enhances GABAergic transmission and inhibits voltage-gated sodium channels [38]	Hepatotoxicity, weight gain, teratogenicity, tremor, sedation [38]
Anti-CGRP monoclonal antibodies (erenumab, fremanezumab, galcanezumab, eptinezumab)	CGRP receptor or ligand inhibitor	Headache	Block calcitonin gene-related peptide or its receptor, reducing neurogenic inflammation and trigeminovascular activation [39]	Injection-site reactions, constipation, hypertension (erenumab); limited data in TBI [39]
Triptans	Serotonin 5-HT1B/1D receptor agonist	Headache	Vasoconstrict cranial arteries and inhibit release of CGRP and substance P [35]	Fatigue, nausea, paresthesia; contraindicated in vascular disease [35]
Botulinum toxin A	Neurotoxin	Nociceptive/Headache	Blocks acetylcholine release at neuromuscular junction; inhibits peripheral and central sensitization [32]	Local muscle weakness, pain at injection site, dysphagia, falls [32]
Interventional procedures (cervical facet, occipital, supraorbital nerve blocks)	Interventional pain therapy	Nociceptive/Neuropathic/ Headache	Interrupt nociceptive input from cervical or cranial nerves, reducing central sensitization and pain chronification [22]	Temporary relief; risk of infection, bleeding, rare nerve injury; may require repeat sessions [22]

## Data Availability

This article is based on previous studies and contains no new studies with human participants or animals performed by any authors.
